# Impurity channels of the long-lived Mossbauer effect

**DOI:** 10.1038/srep15741

**Published:** 2015-10-27

**Authors:** Yao-Yuan Liu, Yao Cheng

**Affiliations:** 1 Department of Engineering Physics, Tsinghua University, Beijing, Haidian, 100084, China

## Abstract

Recent reports have suggested that the nuclear resonant absorption of a long-lived Mossbauer state e.g., ^93m^Nb is mediated by an entangled photon pair (biphoton) rather than by a single photon. Multipolar nuclear excitation in crystals of a single isotope with a natural abundance of 100% spreads in a region containing billions of identical nuclei. As a consequence of the delocalisation, additional decay channels via the impurities, the crystal defects, and the sample boundary, give rise to a density- and temperature-dependent decay. In this letter we report our discovery of impurity channels, the intensity of which is proportional to the square of the ^93m^Nb density.

We recently reported a nonlinear magnetoelectric effect induced by the delocalised nuclear gamma excitation of ^93m^Nb^1^. The magnetoelectric response appeared only with an ac drive and had the characteristics of a magnetoinductance, i.e., the response with a leading phase was stronger at higher drive frequencies. At the end of 2011, many nonlinear resonant peaks were found with driving frequencies of several hundred Hz, which did not depend on applied field, decreased slightly with temperature, and most likely depended on excitation density.

In a previous report^1^ we presented several reasons for identifying the source of the magnetoelectric response as a collective nuclear excitation rather than the orbital electrons of the atom. First, the magnetoelectric response was only found when the ^93m^Nb excitation density was beyond a threshold value of around 10^12^ cm^−3^. Second, the magnetoelectric response was not saturated by a 9 T field at 4 K, because of a small nuclear magneton of ~10^−8^ eV/T^4^,^5^. Third, the magnetoelectric response remained almost unchanged up to 200 K. A weak dependence on temperature revealed stable magnetisation in the crystal, which was not generated by the atomic magneton (~10^−5^ eV/T).

Nuclear resonant absorption without recoil appears when the gamma field is mediated by two entangled photons (a biphoton)^2^,^3^. Due to the multipolar nature of the biphoton, its emission and reabsorption occurs without the ejection of the orbital electrons. This so-called Rabi oscillation^2^ is so fast that the recoil phonon never leaves the delocalised biphoton. The biphoton field absorbs the phonon and obtains a mass when the coupling between the biphoton and the nuclei survives the thermal agitation.

The increasing ratio between Nb Kα and Nb Kβ as reported previously^1^ is attributed to changes in the ratio between the impurity and the boundary channels. The Nb K-lines emitted from the sample surface are free from photoelectric absorption, giving a high Kα/Kβ ratio at the boundary channels. The impurity channels are boosted by the ^93m^Nb density, giving a low Kα/Kβ ratio at the beginning of the measurement.

## Results

**Figure 1:**
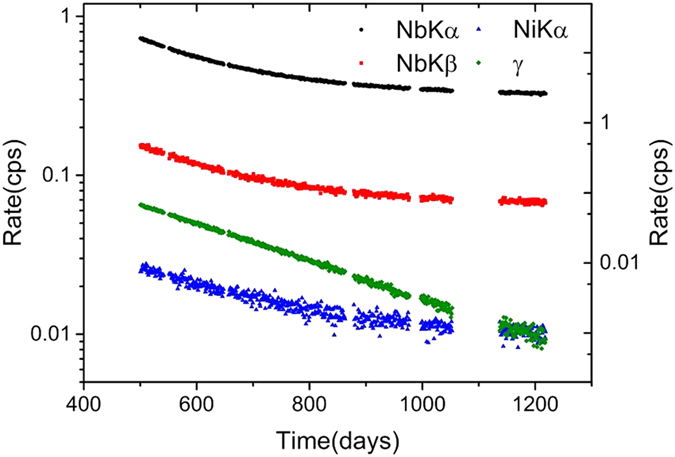
The decays of four x-rays from daily monitoring in cps (counts per second) from 2013 to 2015. The left-hand axis shows the rates of Nb Kα (black dots), Nb Kβ (red squares), and the right-hand axis shows the rates of ^182^Ta γ (green rhombuses), Ni Kα (blue triangles). Day 0 was 10^th^ December 2011 and the monitoring in Beijing started on day 500. The detector cooler failed once at day 864 and recovered at day 879. We recalibrated the spectral energy. Spectral migration due to the instability of the electronic circuits (see Figure S1 in the on-line supplementary material) was considered by selecting channels to give good x-ray development.

Our sample is a high-purity single crystal of niobium^1^. The ^181^Ta impurity became a radioactive isotope of ^182^Ta with a half-life of 114.43 days following neutron irradiation in November 2011. Figure 1 shows the daily count rates of Nb Kα, Nb Kβ, ^182^Ta γ, and Ni Kα from the same detector without any change of measurement regime. These x-ray decays revealed two time constants^1^, apart from the γ emission from ^182^Ta at 67.65 keV. The faster time constant corresponds to the characteristic life-time of ^182^Ta. Decomposition of the fast and slow evolutions revealed that the slow time constant is more than 6 years, revealing a density-dependent decay of ^93m^Nb^1^. We conclude that the contribution of the fast time constant is triggered by the β decay from the ^182^Ta, while the contribution of the slow time constant is trigged by the ^93m^Nb.

**Figure 2:**
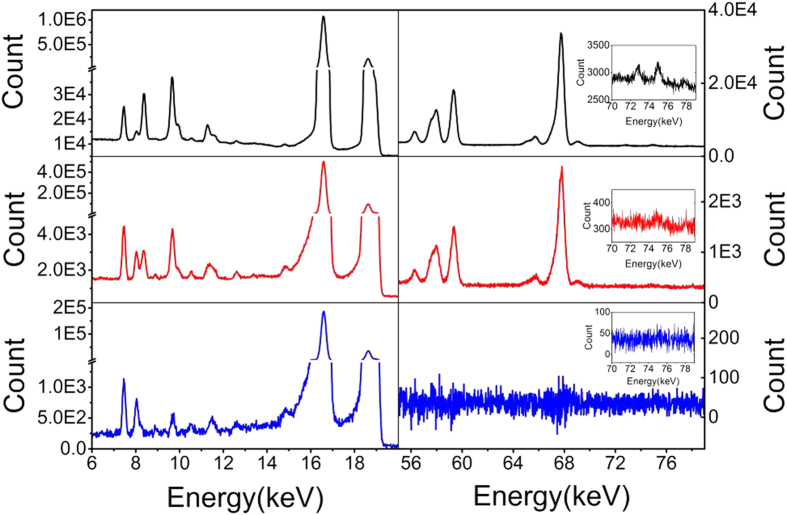
Accumulated x-ray spectra from day 500 to day 863 (black curve), from day 879 to day 1218 (red curve), and the differential mapping (blue curve) obtained using A-0.94 × B, where spectrum A is the cumulative total counts from day 998 to 1218 and spectrum B is the cumulative total counts from day 879 to day 924. The major x-rays in the low-energy section of the black curve show the L-lines of W and Ta (30%). The K-lines of the impurities, e.g., Fe, Ni and Cu, emerge in the red curve. The differential mapping of blue curve removes all the x-ray contributions characterised by the fast time constant of ^182^Ta, which is revealed by the vanishing γ counts at 67.75 keV. The three inset figures show the details around the Pb K-lines. A small peak at 78 keV is contributed by Au Kβ_1_.

Figure 2 shows the x-rays for two ranges of energy of interest. The x-ray energies are listed in the Table in the on-line supplementary material. We have identified the K-lines of the impurities of Fe, Ni, and Cu. Other impurity channels such as those from Mn, Cr, V and Ti, are also being sought. We applied a differential mapping in Fig. 2 to cancel the x-ray contribution from the fast time constant while keeping the x-ray contribution from the slow time constant. The K-lines and L-lines for Ta and W are removed by the differential mapping, which indicates that they are solely contributed by the ^182^Ta. The background-like counts remaining after the differential mapping are ten times higher than the natural background counts. These broad-band x-rays with energies higher than 30.82 keV (the transition energy of ^93m^Nb) contain the contributions of the slow time constant. The readings of ^182^Ta γ in Fig. 1 are slightly contaminated by the contribution of W Kβ, nevertheless they have the same mother nuclide. We applied several rigorous methods to prove that the half-lives of ^182^Ta γ (113.4 ± 0.2 days) and W Kα (113.3 ± 0.3 days) are less than the documented value of 114.43 days. This anomaly indicates that the two decays of ^182^Ta and ^93m^Nb interact slightly, which will be discussed in a later report. The L-lines from Pb and Au are contributed by the Pb and Au in the welding holes of the sample, because the impurity contents of Pb and Au are extremely low in our high-purity sample.

Ni Kα is a pronounced impurity emission that is almost uncontaminated by the W L-lines (see Fig. 2). One β decay of ^182^Ta approximately equates to one decay of Nb K x-rays as estimated using the γ branching ratio, and taking the detection efficiency and sample absorption into account, while ten β decays of ^182^Ta produce one of Ni Kα at day 500. Taking all types of impurity into account, it is clear that the K-shell x-rays from the ppm impurities and from the Nb are of the same order. This allows us to speculate that two electrons from different atoms are simultaneously ejected by the biphoton. The ionised Nb atom becomes an impurity with an orbital hole during a short time interval of the cascade de-excitation. The biphoton field is concentrated at the impurity sites^1^, i.e., the colour centres, producing the major photoelectric effect. The total biphoton energy of 30.82 keV is not sufficient to eject two electrons from the Nb K shell because of the 19-keV binding energy. The biphoton therefore ejects one electron on the “Nb impurity” and the other electron on the impurities nearby, e.g., Ni. The x-ray energy emitted from atoms with multiple ionisation is higher than it is normally, besides which the delocalised photon field dressing^2^ on the impurity atoms changes their atomic energy levels. The up-down zigzags of residual W Kα and W Kβ in the differential mapping of Fig. 2 may reveal an insignificant spectral migration. Clear evidence of the spectral migration is found (see Figure S3 in the on-line supplementary material). The Nb K-lines move towards the low-energy side with each passing day, while the ^93m^Nb density was sufficiently high at day 0. Continued careful inspection free from any detector instability is required in future to reveal whether the time-dependent spectral migration may be attributed to the multiple ionisation or to the dressing photon field.

**Figure 3:**
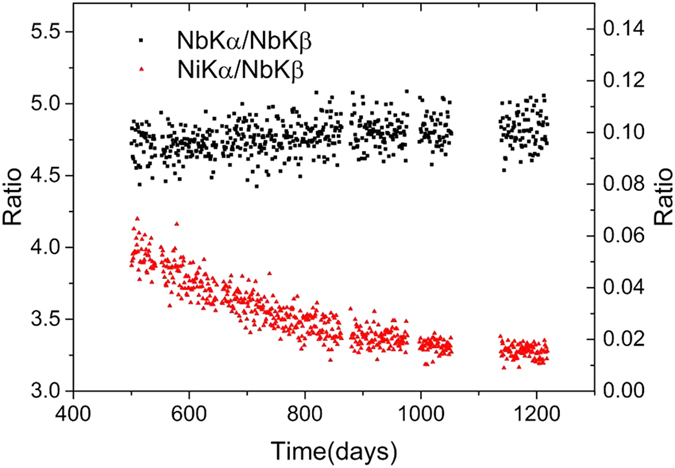
Ratios of the daily counts. The ratio Nb-Kα/Nb-Kβ (left-hand axis and black squares) approached 4.8 from 4.7. The ratio Ni-Kα/Nb-Kβ (right-hand axis and red triangles) shows a quadratic decrease over time.

We show the development of the two ratios Nb-Kα/Nb-Kβ and Ni-Kα/Nb-Kβ in Fig. 3. The time-dependence of the Nb-Kα/Nb-Kβ ratio has already been described in detail^1^. Two boundary channels, on the front and rear sides, reach the active area of the detector with different solid angles. The value of Nb-Kα/Nb-Kβ from the boundary channels is less than its natural value of 5.8 in free space^4^.

We approximated the density of ^93m^Nb N using a linear decay due to the slow time constant. The quadratic ratio of Ni-Kα/Nb-Kβ in Fig. 3 gives an N^2^-dependency of the impurity channels, as speculated previously^1^. This quadratic ratio also reveals the important fact that the Ni is located inside the crystal that constitutes the impurity. In contrast, Au and Pb outside the crystal give a linear response to the sample radiation.

## Discussion

The physics of impurity channel is rather subtle. The standard deviation 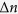 of the Poissonian photon statistics is 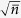, where 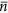 is the expected photon count. The fluctuation 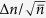 of Ni Kα is 0.82 ± 0.12, as evaluated between day 998 and day 1218. It is noteworthy that more than 99% of the Ni Kα from Ni atoms are randomly absorbed by the sample. 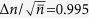 gives rise to an uniform photon train without any fluctuation emitted from the impurity sites, which are randomly distributed in the Nb crystal. Furthermore, the pace of the photon train follows the N^2^-rule of the ^93m^Nb density in the entire reservoir, which is released by the random decays of ^182^Ta and ^93m^Nb. To explain this paradox, the co-emission of Nb and Ni x-rays from the boundary channel dominates after day 998, when the activity of ^182^Ta inside the crystal has become sufficiently weak. Even supposing that we adopt this interpretation of the boundary channel, the antibunched^2^ photon statistics are still somewhat puzzling, however. A regular x-ray source will give a fluctuation 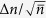 of 0.99 instead of 0.82, because our detector only has a 2% solid angle for receiving the isotropic photons.

X-rays from impurities were mainly found for energies less than 15.4 keV, i.e., half the ^93m^Nb energy. No contributions from Zn, Zr, Mo, or Ta with the slow time constant were found. The 30-keV energy of ^93m^Nb is sufficient to eject a combination of one Nb K electron and one Ta L electron. The binding energy of the Ta L_3_-shell is 9.88 keV, which deviates from the binding energy of the Cu K-shell (8.98 keV) by less than 1 keV. There is no reason why electrons would be ejected at the minor impurities while the major impurity of the Ta remained untouched. The missing Ta L-lines and the perfect photon antibunching^2^ will doubtless play an important role in our understanding of the underlying physics in any future investigation.

## Conclusions

The β decay of ^182^Ta^5^ releases no Ta x-rays, unless an additional decay channel of ^182^Ta opens to emit a neutron. The extra neutron decay from ^182^Ta gives rise to a shorter half-life than normal. The impurities are thus in interaction with the delocalised ^93m^Nb. The N^2^ relationship of Ni-Kα/Nb-Kβ presented in Fig. 3 provides ample evidence of the impurity channel. It also provides evidence for the cooperative ejection of two electrons by the biphoton.

## Additional Information

**How to cite this article**: Liu, Y.-Y. and Cheng, Y. Impurity channels of the long-lived Mossbauer effect. *Sci. Rep.* **5**, 15741; doi: 10.1038/srep15741 (2015).

## Supplementary Material

Supplementary Information
