# Supplementary material for: Impurity channels of the long-lived Mossbauer effect
Source: Sci Rep. 2015 Oct 27;5:15741. doi: 10.1038/srep15741 (PMC4621536; doi:10.1038/srep15741)
Supplement: Supplementary Information [file srep15741-s1.pdf]

# Supplementary Information

# Impurity channels of the long-lived Mossbauer effect

Yao-Yuan Liu and Yao Cheng*
Department of Engineering Physics, Tsinghua University, Beijing, Haidian, 100084, China
*Corresponding Author: Yao Cheng

**Methods**

The details of sample preparation have been reported in reference 1. The sample is a single-crystal of Nb (an oval plate, 1.2 mm × 12 mm ×13 mm), using neutron activation[5] at $2\times10^{12}$ n cm$^{-2}$ s$^{-1}$ for 5 hours, in the reactor at Tsinghua University, Hsinchu, in 2008 and again in November 2011. Most of the thermal neutrons were removed by a Cd envelope, so that only fast neutrons with a flux of $10^{11}$ n cm$^{-2}$ s$^{-1}$ passed through the sample. We drilled six holes in the sample and wired Cu leads to the sample using Indium solder for the magnetoelectric measurement[1] prior to the second activation in 2011. The activation of the residual Indium solder was observed in holes following the second activation. One year later in 2012, we roughly cleaned the residual Indium solder and filled the holes with gold rods for the electrical connections in order to repeat the magnetoelectric measurements[1]. The gold rods remained in the sample holes, as does the contamination of the indium solder.

Two silicon detectors (A and B) of the same type, manufactured by the same company AMPTEK, were used. Detector A is located in Hsinchu and Detector B is located in Beijing. The daily monitoring of x rays from day 0 (10$^{th}$ December 2011) to day 174 were made using Detector A in Hsinchu, Taiwan, with the remainder being made using Detector B in Beijing. The detection efficiency of Nb Kα is about 10% higher than the detection efficiency of Nb Kβ. The active area of the applied silicon detector is 25 mm$^2$. The active element of the detector is located about 5 mm from the sample. We calibrated the x-ray energy of Detector A in Taiwan using the L-lines from a W target under a 30-keV E-beam. The calibration of the x-ray energy for Detector B was achieved thanks to the acquired knowledge obtained from the measured x-rays of the two detectors.

The low-temperature experiments undertaken to measure the magnetoelectric effect[1] were carried out in three different periods. The first cooling period lasted one week and began ten days before we started this 4-year monitoring programme in 2011. The second cooling period lasted 40 days from day 90 to day 130 of the same programme in 2012. The third cooling period took place between day 350 and day 500. Seven low-temperature measurements were made by cooling our sample at 4 K for 24 hours each time, otherwise it was stored at room temperature.

**Detector stability**

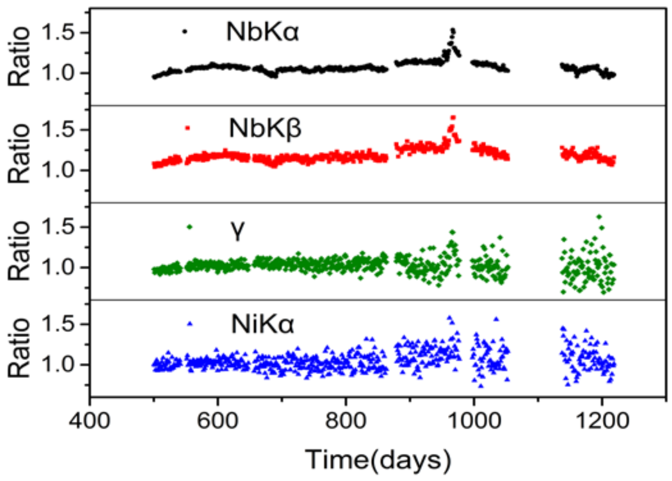


**Figure S1.** The spectral migrations of four x-rays, Nb Kα (black dots), Nb Kβ (red squares), $^{182}$Ta γ (green rhombuses), Ni Kα (blue triangles). The gross counts without removing their background counts are separated by their energies into two parts, i.e. left-half peak and right-half peak. The development of their ratios (left-half/right-half) shows a false spectral migration that mainly depends on the temperature of the detector electronics, as shown in the Fig. S2 of this on-line supplementary. We don't know yet the reason of a significant spectral shift at day 967, although the detector temperature was constant at 228 K the whole time. The abnormal data from day 953 to day 977 are ignored in the evaluation of the differential mapping of Fig. 2 and the half-lives of $^{182}$Ta γ and W K$\alpha_1$ in the main text. Assuming the energy of $^{182}$Ta γ and Ni Kα are correct, the energy of Nb Kα (Nb Kβ) are 5.9±1.2 (7.4±0.9) eV higher than the documented values, as evaluated by the cumulative total counts from day 500 to day 863.

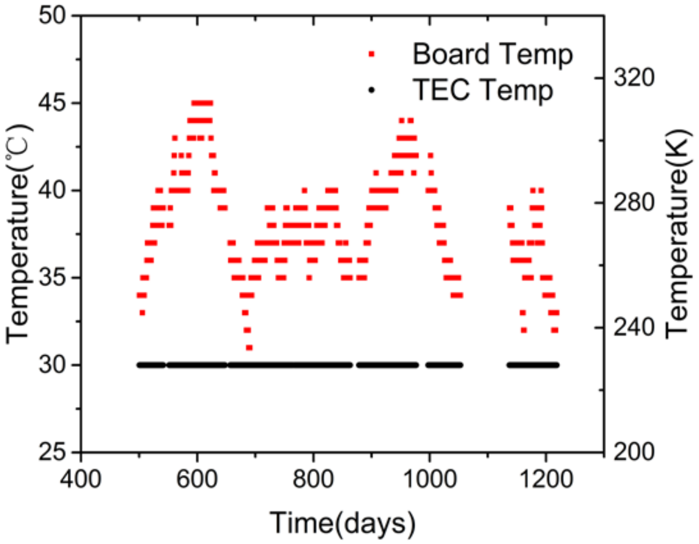


**Figure S2.** Temperature monitoring of Detector B, i.e. board temperature for the electronic circuit (red squares) and TEC temperature for the active area (black dots).

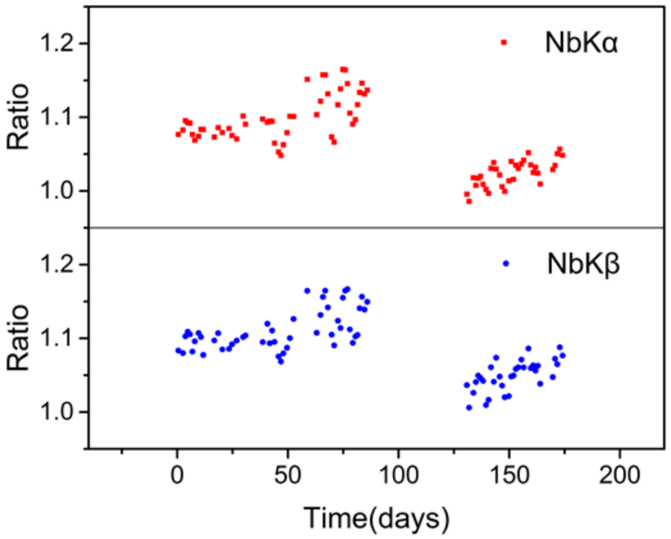


**Figure S3.** The true spectral migrations of two major x-rays, Nb Kα (red squares), Nb Kβ (blue dots), evaluated with the same method of Fig. S1 in this on-line supplementary. These data are measured in Taiwan using Detector A from day 0 to day 174. The sample was stored at 4 K to test the temperature effect between day 90 and day 130. Two x rays moved toward the low-energy side within a month, while the temperatures of detector were stable, as shown in Fig. S4 in this on-line supplementary. The calibration carried out with the circuit board temperature of 30 °C in Taiwan (2011) shows that the energy of Nb Kα (Kβ) emitted from the delocalised $^{93m}$Nb are higher by 12.0±4.3 (15.4±4.7) eV than the documented values, as evaluated by the cumulative total counts from day 131 to day 174. The increase of ratio from 1.0 to 1.1 corresponds to a 10-eV spectral shift towards the low-energy side.

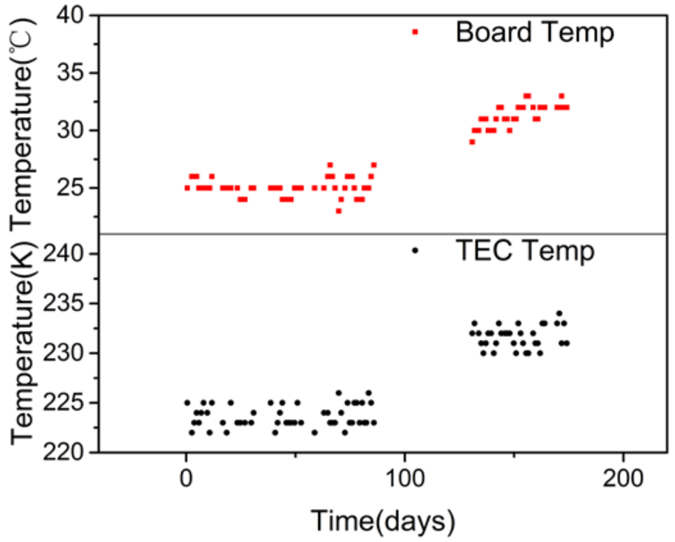


**Figure S4.** Temperature monitoring of Detector A from day 0 to day 174, i.e., board temperature for the electronic circuit (red squares) and TEC temperature for the active area (black dots).

**Table :** The detected x-ray energies[4] of interest expressed in keV.

| | | | | | | |
|---|---|---|---|---|---|---|
| X rays | Fe Kα | Ni Kα | Cu Kα | Ni Kβ | Cu Kβ | Au Lα |
| Energy | 6.47 | 7.47 | 8.04 | 8.25 | 8.91 | 9.67 |
| X rays | Pb Lα | Au Lβ | Pb Lβ | Pb Lγ | Nb Kα | Nb Kβ |
| Energy | 10.50 | 11.51 | 12.61 | 14.77 | 16.58 | 18.62 |
| X rays | Ta Lα | W Lα | Ta Lβ | W Lβ | Ta Lγ | W Lγ |
| Energy | 8.15 | 8.40 | 9.34 | 9.67 | 10.90 | 11.29 |
| X rays | Ta $K\alpha_2$ | Ta $K\alpha_1$ | W $K\alpha_2$ | W $K\alpha_1$ | Pb $K\alpha_2$ | Pb $K\alpha_1$ |
| Energy | 56.28 | 57.54 | 57.98 | 59.32 | 72.81 | 74.97 |
| X rays | $^{182}$Ta γ | $^{182}$Ta γ | W $K\beta_1$ | W $K\beta_2$ | W $K\beta_3$ | Au $K\beta_1$ |
| Energy | 65.71 | 67.75 | 67.24 | 69.07 | 66.95 | 77.98 |
